# The Cost Effectiveness of Donafenib Compared With Sorafenib for the First-Line Treatment of Unresectable or Metastatic Hepatocellular Carcinoma in China

**DOI:** 10.3389/fpubh.2022.794131

**Published:** 2022-03-31

**Authors:** Rui Meng, Yingdan Cao, Ting Zhou, Hongfei Hu, Yijin Qiu

**Affiliations:** School of International Pharmaceutical Business, China Pharmaceutical University, Nanjing, China

**Keywords:** cost-effectiveness, hepatocellular carcinoma (HCC), donafenib, sorafenib, first-line treatment, China

## Abstract

**Background:**

Recent clinical trials have demonstrated that donafenib has superior efficacy and safety compared with sorafenib in Chinese patients with unresectable or metastatic hepatocellular carcinoma (HCC). The objective of this study was to assess the cost effectiveness of donafenib compared with sorafenib for the treatment of patients with unresectable or metastatic HCC in China.

**Methods:**

A three-state partitioned survival model was developed to perform a cost-effectiveness analysis comparing donafenib and sorafenib from a Chinese healthcare payer's perspective. The model adopted a lifetime horizon and a 4-week cycle length. Survival data were derived from the ZGDH3 study and fitted with standard parametric functions for extrapolation beyond the trial period. Cost data were obtained from the mean price of publicly listed online bids in 2021 and medical service prices across provinces in China. Utility data were obtained from previous literature. The cost and health outcomes were discounted at an annual rate of 5%. Deterministic and probabilistic sensitivity analyses (PSAs) were carried out to verify the robustness of the model.

**Results:**

Compared with sorafenib, donafenib incurred a higher cost (US$22,330.23 vs. US$14,775.92) but yielded more quality-adjusted life years (1.045 vs. 0.861 QALYs). The incremental cost-effectiveness ratio (ICER) for donafenib was US$41,081.52 per QALY gained (ICER = US$13,439.10/QALY). The PSA results indicated that at a willingness-to-pay threshold of 3 times the GDP in China, the probability of donafenib being cost effective was 16.9%. The ICER (US$13,439.10/QALY) decreased when the branded price of sorafenib was used in the model.

**Conclusions:**

Donafenib is unlikely to be cost effective compared with sorafenib for the first-line treatment of unresectable or metastatic HCC in China. Reducing the price of donafenib can increase the possibility of it being cost effective in the future.

## Introduction

In 2020, liver cancer was the sixth most common type of cancer globally, with 905,677 new cases, and the third leading cause of cancer-related death, taking the lives of 830,108 individuals. In China, it was the fifth most commonly diagnosed cancer, accounting for 410,038 new cases, and the secondary leading cause of cancer-related death, with 391,152 individuals affected (1). Hepatocellular carcinoma (HCC) accounts for approximately 90% of all liver cancer cases (2), and the main factors affecting the incidence of HCC in China is chronic hepatitis B (HBV) infection and aflatoxin-contaminated foods (3). Most HCC patients are diagnosed at a time when the tumors have progressed or metastasized, so radical surgical treatment cannot be administered; therefore, drug therapy has become an important treatment strategy for these patients (4).

One randomized, open-label, parallel-controlled multicenter phase II-III trial (ZGDH3) enrolled 668 patients with unresectable or metastatic HCC, a Child-Pugh score ≤ 7, and no prior systemic therapy to investigate the efficacy and safety of donafenib vs. sorafenib as a first-line treatment of unresectable or metastatic HCC (5). Donafenib is an orally administered small molecule multikinase inhibitor that exhibits anti-tumor properties and has multiple targets; it can inhibit not only the activity of multiple tyrosine kinases, including vascular endothelial growth factor receptor and platelet-derived growth factor receptor, but also various Raf kinases and the downstream Raf/MEK/ERK signal transduction pathway, thereby suppressing tumor cell proliferation and angiogenesis (6). This pivotal study showed the superiority of donafenib over sorafenib in Chinese patients with advanced HCC, leading to the significant prolongation of overall survival (OS) (5). In the full analysis set (FAS), the median OS was 12.1 months in the donafenib arm and 10.3 months in the sorafenib arm, with a hazard ratio (HR) of 0.831. In the intention-to-treat population, the mOS outcomes with donafenib were significantly longer than those with sorafenib (12.0 months vs. 10.1 months; HR, 0.839). Nevertheless, there was no significant difference between the two groups in terms of the secondary efficacy endpoints of median progression-free survival 3.7 months vs. 3.6 months), objective response rate (4.6 vs. 2.7%), and disease control rate (30.8 vs. 28.7%). In the safety analysis, patients treated with donafenib had significantly fewer drug-related grade 3 or higher serious adverse events (AEs) than patients receiving sorafenib (38 vs. 50%).

Based on the results of the ZGDH3 study (5), donafenib was approved in 2021 by the National Medical Products Administration (NMPA) of China for the first-line treatment of patients with unresectable HCC who had not received systemic treatment in the past. Before the approval of donafenib, Chinese clinical guidelines recommended sorafenib, lenvatinib, FOLFOX4 chemotherapy, and atezolizumab plus bevacizumab as first-line systemic treatment for patients with advanced unresectable HCC in China (7). Sorafenib and lenvatinib are covered by China's national health insurance, and the price was sharply reduced via price negotiation with the Chinese government in 2017 and 2020, respectively (8, 9). In addition, the price of sorafenib was further reduced due to Volume-based Procurement (VBP) in 2021; therefore, sorafenib has become the most commonly used standard first-line treatment for advanced HCC in China.

Although donafenib has demonstrated better clinical efficacy than sorafenib, there is a lack of economic evidence in support of this new drug. This study aimed to evaluate the cost effectiveness of donafenib compared with sorafenib for the first-line treatment of patients with unresected HCC in China to better inform clinical decision-making related to medical insurance catalog access and to provide more evidence for the rational clinical use of drugs.

## Materials and Methods

### Model Structure

**Figure 1:**
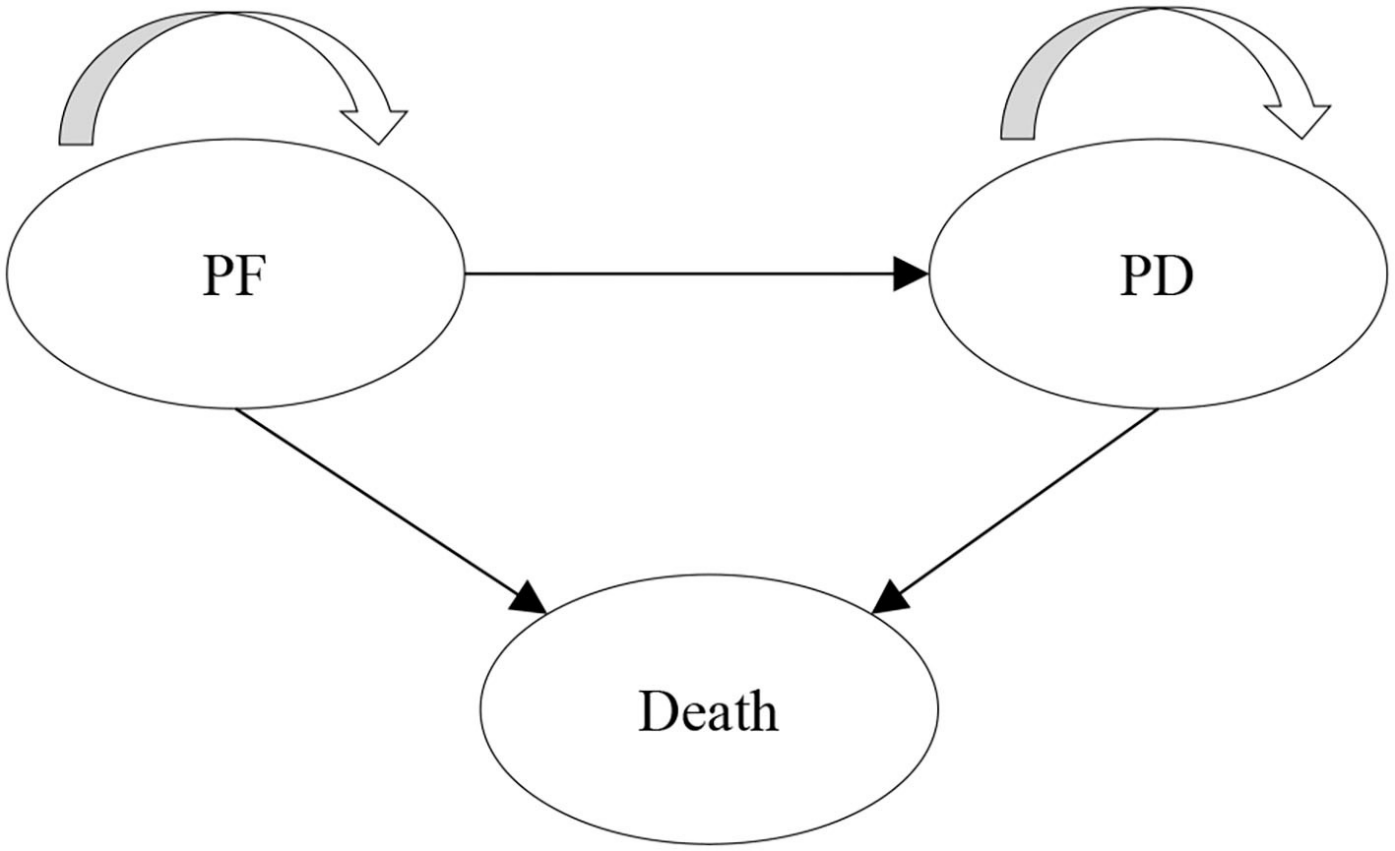
Model structure. PF, progression-free; PD, progressive disease.

A partitioned survival model (PSM) was developed to evaluate the long-term cost and health outcomes of donafenib vs. sorafenib for the treatment of HCC. PSM estimates the proportion of patients in each health state through a series of independently modeled, non-mutually exclusive survival curves and is commonly used for the economic evaluation of anti-tumor drugs (10). Three health states were included in the model: progression-free (PF), progressive disease (PD), and death, which are consistent with the previous model submitted to the National Institute for Health and Care Excellence (NICE) (11). All patients initially entered the PF state. After treatment, the patient could stay in the PF state, progress to the PD state or die, and the patients in the PD state could stay in this state or die. The model structure is shown in Figure 1.

A lifetime horizon was applied to capture the full impact of the intervention on the cost and health outcomes of HCC patients from a Chinese healthcare payer's perspective. The cycle length was set to 4 weeks (28 days) to be consistent with the medication cycle in the ZGDH3 study (5). The outcome included total costs, quality-adjusted life years (QALYs), life years, and the incremental cost-effectiveness ratio (ICER). According to the China Guidelines for Pharmacoeconomic Evaluations in 2020 (12), we adopted an annual discount rate of 5% for cost and health outcomes (QALYs). The model was constructed using Microsoft Excel 2019.

### Patient Treatment Pathways

The characteristics of the target population in this model were assumed to be the same as those in the ZGDH3 study (5), comprising a hypothetical cohort of 1,000 patients who had unresectable or metastatic HCC and had not received systemic chemotherapy. Patients received oral donafenib (0.2 g twice a day) and sorafenib (0.4 g twice a day) until the disease progressed or severe toxicity or intolerance occurred. Patients who failed first-line treatment received subsequent treatment until death.

### Efficacy

The clinical efficacy parameters of the donafenib and sorafenib groups were derived from the Kaplan-Meier (KM) curves of the ZGDH3 study (5). In addition, extrapolation was required in the cost-effectiveness analysis due to the limited follow-up time of the KM curves in clinical trials. GetData Graph Digitizer (version 2.26; http://www.getdata-graph-digitizer.com/download.php) software was used to pick points from the PFS curve and the OS curve to obtain the overall survival rate and progression-free survival rate of each cycle node during the trial period. The method recommended by Guyot et al. (13) was used to reconstruct individual patient data, and six types of standard parameter distributions were used to fit and extrapolate the OS and PFS curves, including exponential, Gompertz, Weibull, log-logistic, lognormal and gamma distributions. The Akaike information criterion (AIC) and Bayesian information criterion (BIC) were applied to choose the best fitting distribution. The lower the AIC and BIC values are, the better the goodness of fit. The proportion of patients in each state beyond the trial period of the donafenib arm and sorafenib arm was calculated by the survival function formula of the optimal distribution. The AIC and BIC of different distributions of restructure curves are shown in Supplementary Table S1. The survival function formula and parameters of the best fitting distribution of the KM curve are shown in Supplementary Table S2. Based on AIC and BIC, the lognormal distribution was chosen to simulate the OS curve of donafenib and sorafenib and the PFS curve of donafenib. In addition, log-logistic distribution was used to reconstruct the PFS curve of sorafenib. The median OS and PFS of reconstructed KM curves were similar to those presented in the ZGDH3 study (Supplementary Table S3).

### Cost

This study adopted Chinese healthcare payer's perspective; therefore, only direct medical costs were considered, including the drug acquisition cost, administration cost, cost of follow-up visit, cost of subsequent treatment after disease progression, cost of management of adverse events, and end-of-life cost. In terms of the drug acquisition cost, the market price of donafenib was US$29.95/100 mg in 2021 (14), and the cost of medication per cycle was US$3,354.38. The manufacturer of donafenib provides a Patient Assistance Program (PAP) in China; thus, the actual cost after donation was calculated in this model (15). The price of sorafenib in China dropped sharply in 2021 due to VBP. In this study, the mean VBP price of sorafenib was applied, which was US$3.31/200 mg and US$370.36 per cycle (16) (Table 1).

**Table 1:** Model parameters.

**Model inputs**	**Value**	**SE**	**Distribution**	**Alpha**	**Beta**	**Minimum**	**Maximum**	**Source**
**Cost**								
**Drug acquisition per cycle**								
Donafenib	3354.38	256.71	Gamma	170.74	19.65	2348.06	3354.38	(14)
Sorafenib	370.36	301.26	Gamma	1.51	245.05	361.11	1542.06	(16)
**Administration**								
Diagnosis and examination	1.45	0.96	Gamma	2.27	0.64	0.58	4.35	The mean price of
**Follow up per visit (PF/PD)**								medical services in
Routine blood test	1.45	0.48	Gamma	9.09	0.16	0.87	2.75	12 provinces and
Liver function test	5.80	1.74	Gamma	11.13	0.52	2.75	9.57	cities
Thyroid function	8.70	1.77	Gamma	24.01	0.36	6.38	13.33	
Fasting glucose	0.72	0.18	Gamma	16.67	0.04	0.58	1.28	
Electrolytes	6.52	0.67	Gamma	96.04	0.07	4.93	7.54	
Renal function	5.36	1.07	Gamma	25.01	0.21	3.77	7.97	
Coagulation	5.80	1.50	Gamma	14.99	0.39	2.83	8.70	
Routine urinalysis	0.58	0.37	Gamma	2.46	0.24	0.14	1.59	
12-lead electrocardiography	3.19	0.59	Gamma	29.05	0.11	2.90	5.22	
Alpha fetoprotein	2.32	0.70	Gamma	10.90	0.21	1.59	4.35	
Color Doppler echocardiography	17.39	4.99	Gamma	12.14	1.43	9.42	28.99	
CT	21.74	15.16	Gamma	2.06	10.57	13.04	72.47	
**Subsequent treatment (PD) per cycle**								
Cost of subsequent treatment	959.16	97.87	Gamma	96.04	9.99	767.33	1150.99	(17)
**Management of adverse events**								
Hand foot skin reactions	12.97	1.32	Gamma	96.04	0.14	10.38	15.57	(18)
Hypertension	35.46	3.62	Gamma	96.04	0.37	28.36	42.55	(19)
Elevated AST	56.54	5.77	Gamma	96.04	0.59	45.23	67.84	(19)
Hypophosphatemia	42.93	11.42	Gamma	14.13	3.04	7.16	51.94	(19)
**End of life**	1870.00	190.82	Gamma	96.04	19.47	1496.00	2244.00	(20)
**Efficacy parameter**								
μDONOS	2.5312		Lognormal	-	-	-	-	(5)
δDONOS	1.0034		Lognormal	-	-	-	-	(5)
μSOROS	2.3764		Lognormal	-	-	-	-	(5)
δSOROS	0.9444		Lognormal	-	-	-	-	(5)
μDONPFS	1.3364		Lognormal	-	-	-	-	(5)
δDONPFS	0.8439		Lognormal	-	-	-	-	(5)
λSORPFS	3.2420		Log-logistic	-	-	-	-	(5)
γSORPFS	2.2093		Log-logistic	-	-	-	-	(5)
**Utility**								
PF	0.745	0.008	Beta	2267.02	775.96	0.730	0.760	(11)
PD	0.678	0.012	Beta	1062.37	504.55	0.655	0.701	(11)
**Disutility of adverse events**								
Hand foot skin reactions	0.116	0.012	Beta	84.78	646.11	0.093	0.139	(21)
Hypertension	0.012	0.001	Beta	94.88	7811.42	0.010	0.014	(22)
Elevated AST	0.000	-	Constant	-	-	-	-	(23)
Hypophosphatemia	0.181	0.018	Beta	78.48	355.09	0.145	0.217	(24)
**Risk of adverse event**								
Hand foot skin reactions associated with donafenib	0.057	0.006	Beta	90.50	1494.42	0.046	0.069	(5)
Hypertension with associated donafenib	0.090	0.009	Beta	87.30	881.59	0.072	0.108	(5)
Hand foot skin reactions associated with sorafenib	0.124	0.013	Beta	84.06	596.56	0.099	0.148	(5)
Hypertension associated with sorafenib	0.087	0.009	Beta	87.57	915.51	0.070	0.105	(5)
Elevated AST associated with sorafenib	0.048	0.005	Beta	91.36	1804.13	0.039	0.058	(5)
Hypophosphatemia associated with sorafenib	0.045	0.005	Beta	91.65	1936.08	0.036	0.054	(5)
**Discount rate**	0.050	-	Constant	-	-	0.000	0.080	(12)

*SE, standard error; PF, progression-free; PD, progressive disease; CT, computed tomography; AST, aspartate aminotransferase; DON, donafenib; SOR, sorafenib; OS, overall survival; PFS, progression-free survival*.

The cost per follow-up visit included the cost of laboratory testing and computed tomography (CT). The follow-up cost was obtained from the mean price of medical services in 12 provinces across China to represent the national medical service price level (i.e., Beijing, Shanghai, Jiangsu, and Zhejiang). Based on the ZGDH3 study, follow-up visits were completed every 4 weeks, and an imaging evaluation was performed every 8 weeks. After disease progression, follow-up visits were completed every 8 weeks (5).

Patients in both groups were assumed to receive the same subsequent treatment after disease progression. The model considered adverse events to be grade 3 or higher, and the incidence was not less than 5% in the ZGDH3 study, including hand foot skin reactions, hypertension, elevated aspartate aminotransferase (AST) and hypophosphatemia. In addition, we considered the end-of-life cost for patients. The cost of subsequent treatment, management of adverse events, and end-of-life cost were derived from the published literature (17–20). All costs were adjusted to US dollars in 2020 (1RMB = 0.14493 US dollars).

### Health Utility

The health state utilities were obtained from NICE TA551 (11), which was measured with the EQ-5D-3L for advanced HCC patients treated with lenvatinib and sorafenib. The utility of PFS and PD states were 0.745 and 0.678, respectively. The occurrence of adverse events due to sorafenib and lenvatinib might have an impact on patients' health-related quality of life. This study also considered the patients' disutility due to adverse events, which were obtained from previous studies (21–24) (Table 1).

### Cost-Effectiveness Analysis

When conducting the base-case analysis, the willingness-to-pay (WTP) threshold was set at 1–3 times the gross domestic product (GDP) per capita of China in 2020 (US$10,499.74–31,499.23/QALY). We conducted one-way deterministic sensitivity analyses (DSA) to assess the robustness of ICER with respect to the change in key parameter estimates and assumptions. The parameter variation range of the DSA was derived from the 95% confidence interval reported in the relevant literature or assumed to be a variance of ±20% of the base-case value when such data were not available.

Since donafenib was only recently approved, there may be much room for price reductions in the future. Therefore, the minimum cost of donafenib was set to a 30% decrease from the base-case value, and the maximum value was still the current market price. The price range of sorafenib was based on the variation in VBP prices in 2021, and the cost range of administration and follow-up visits were determined from the variation in medical services in 12 provinces. A probabilistic sensitivity analysis (PSA) was performed based on the values sampled from the distributions of model inputs, and all efficacy parameters were simulated through Cholesky decomposition. A total of 1,000 iterations of ICER (Monte Carlo simulation) estimation were presented in the cost-effectiveness plane and cost-effectiveness acceptability curve for donafenib vs. sorafenib. The cost parameters followed a gamma distribution, and the utility and the incidence of adverse events followed a beta distribution (Table 1).

We also conducted two scenario analyses to explore the impact of different clinical situations on the economic results: (a) To eliminate the uncertainty caused by the extrapolation of the KM curve, we only used the survival data derived from the original KM curve during the trial period to estimate the ICER. (b) Due to policy influences in China, the price of sorafenib varies between different manufacturers, so the mean VBP price of sorafenib was used in the base-case analysis. However, the price of branded sorafenib was more expensive; taking this into account, we used the price of the branded drug (US$13.77/200 mg) to estimate the ICER to more comprehensively assess the impact of different prices of sorafenib on the results.

## Results

### Base-Case Analysis

In the base-case analysis, compared with sorafenib, donafenib was more costly (US$22,330.23 vs. US$14,775.92) but yielded more QALYs (1.045 vs. 0.861) and life years (1.502 vs. 1.239). The ICER was far more than US$10,499.74–31,499.23/QALY, which suggested that at the WTP threshold of China, donafenib was not cost effective compared to sorafenib (Table 2).

**Table 2:** Base-case and scenario analysis results.

		**Cost**	**QALY**	**LYs**	**ICER**
**Base case**					
	Donafenib	US$22,330.23	1.045	1.502	US$41,081.52/QALY
	Sorafenib	US$14,775.92	0.861	1.239	-
**Scenario 1**					
	Donafenib	US$15,830.60	0.774	1.100	US$61,832.12/QALY
	Sorafenib	US$11,291.53	0.701	0.999	-
**Scenario 2**					
	Donafenib	US$22,330.23	1.045	1.502	US$13,439.10/QALY
	Sorafenib	US$19,858.97	0.861	1.239	-

*QALY, quality-adjusted life-year; Lys, life years; ICER, incremental cost-effectiveness ratio*.

### Sensitivity Analysis

**Figure 2:**
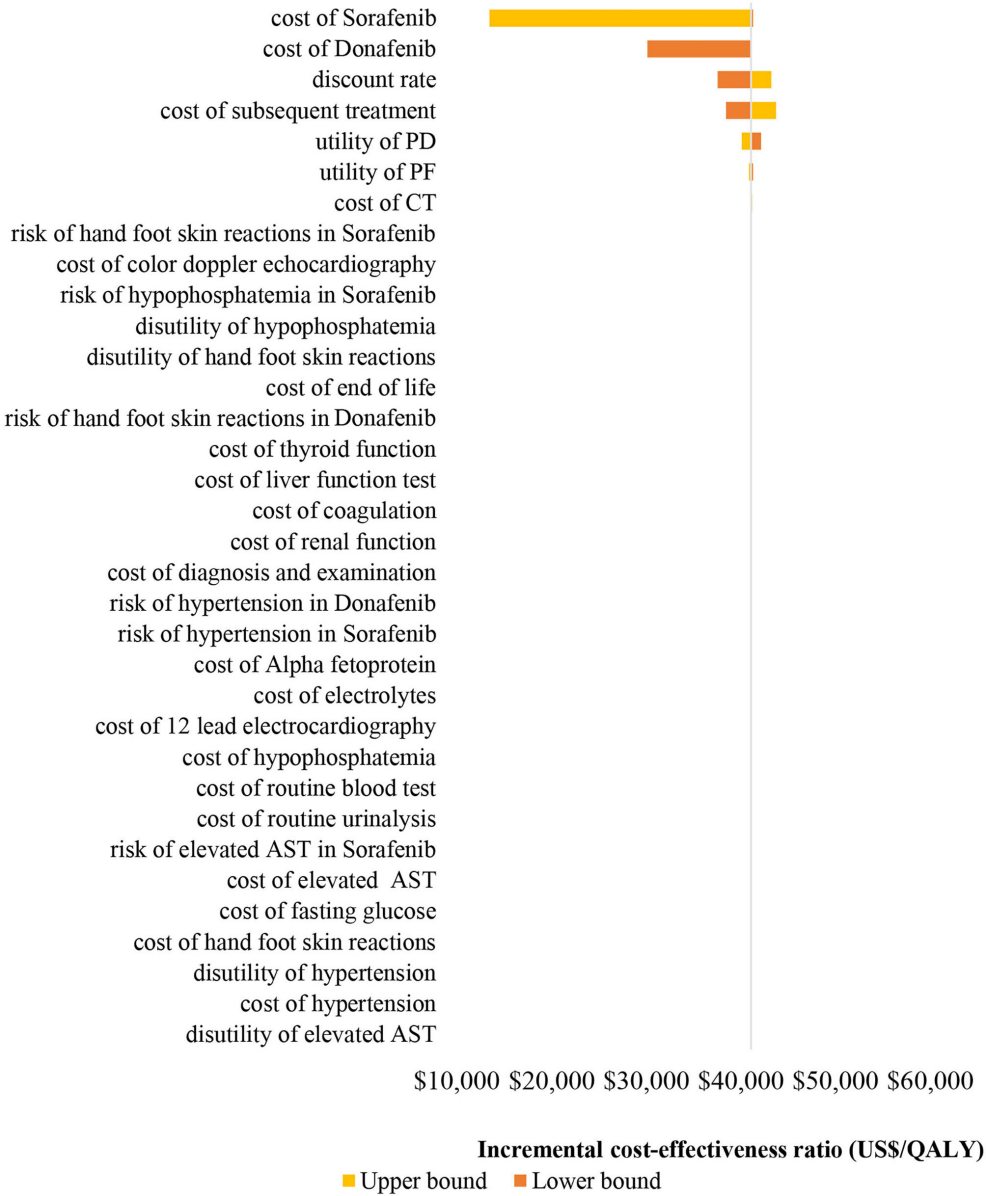
Tornado diagram of the one-way deterministic sensitivity analyses. PF, progression-free; PD, progressed disease; CT, computed tomography; AST, aspartate aminotransferase; QALY, quality-adjusted life-year.

**Figure 3:**
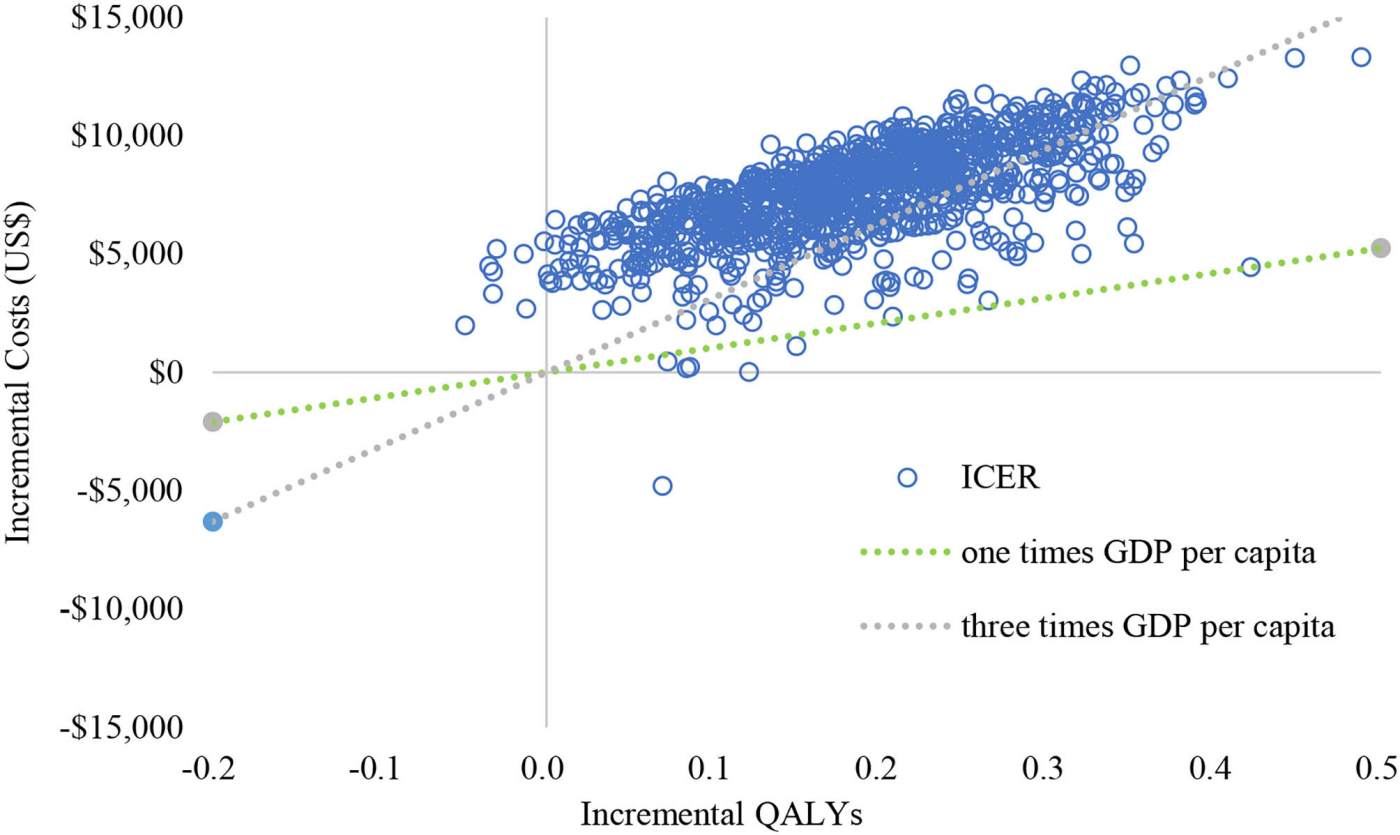
Cost-effectiveness plane of the 1,000 Monte Carlo simulation runs. GDP, gross domestic product; QALY, quality-adjsted life-year; ICER, incremental cost-effectiveness ratio.

**Figure 4:**
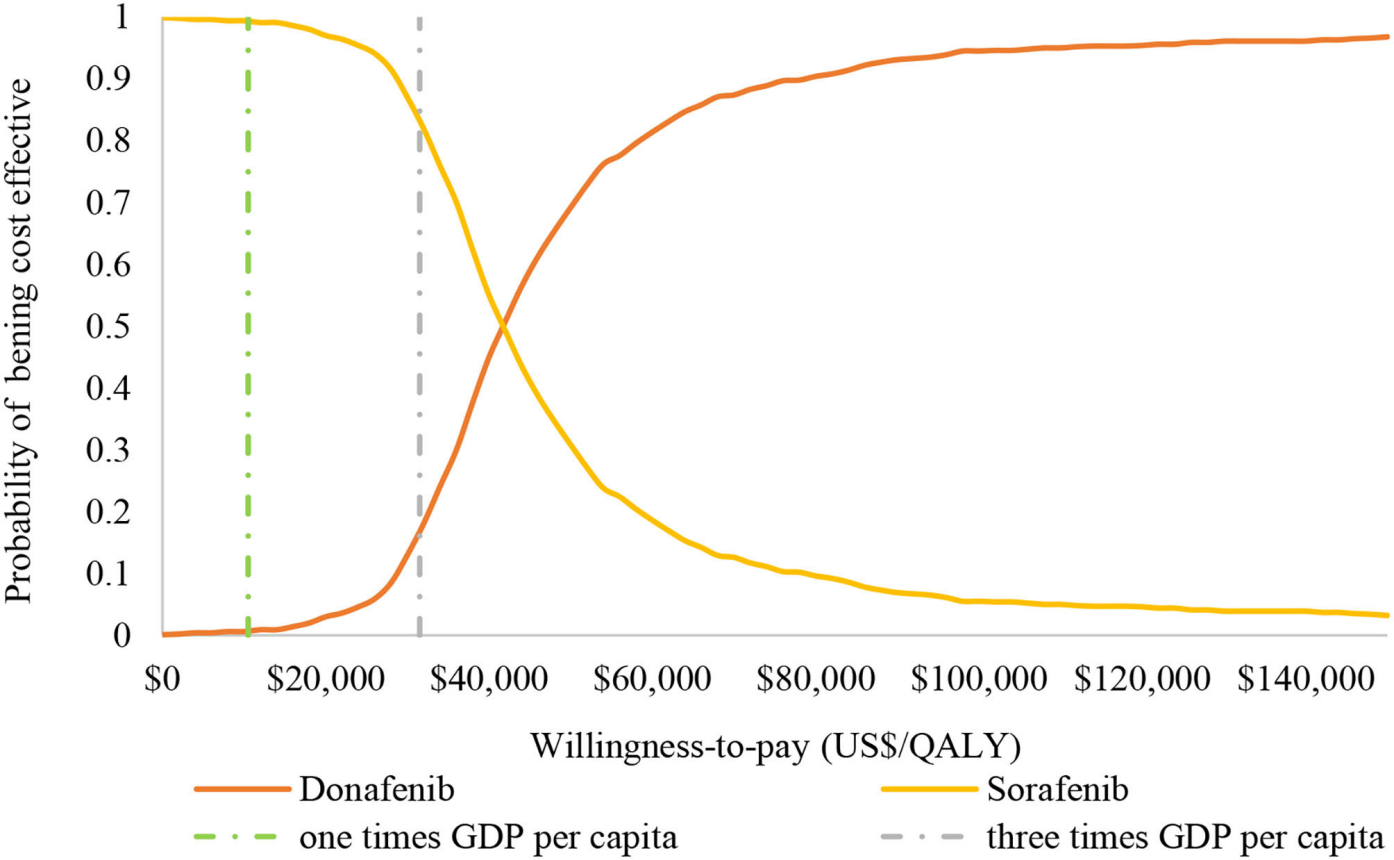
Cost-effectiveness acceptability curve of donafenib vs. sorafenib. GDP, gross domestic product; QALY, quality-adjusted life-year.

The results (tornado diagram) of the DSA suggested that the parameters having the greatest impact on ICER were the cost of sorafenib and donafenib, discount rate, utility of PF and PD state (Figure 2). The PSA results showed that most ICERs were located in the southeast quadrant, indicating that in most cases, compared with sorafenib, donafenib was more costly but led to more QALYs (Figure 3). The cost-effectiveness acceptability curve (CEAC) suggested that under the WTP threshold of 1–3 times the per capita GDP of China (US$10,499.74–31,499.23/QALY), the probability of donafenib being cost effective was 6–16.9%, which verified the robustness of the base-case analysis results (Figure 4).

### Scenario Analysis

The results of the scenario analysis are presented in Table 2. When only the survival data derived from the original KM curve without extrapolation was applied to the model (Scenario a), the result was consistent with the base-case analysis; donafenib was more costly (US$15,830.60 vs. US$11,291.53) but yielded more QALYs (0.774 vs. 0.701) and life years (1.100 vs. 0.999) than sorafenib. The ICER was US$61,832.12/QALY, which suggested that donafenib was not cost effective compared to sorafenib. When we used the branded price of sorafenib (US$13.77/200 mg, Scenario b), the result was opposite to that obtained in the base-case analysis; the ICER was US$13,439.10/QALY, which suggested that donafenib was more cost effective than sorafenib.

## Discussion

The main findings of base-case analysis indicated that compared with sorafenib, donafenib was not cost effective for first-line treatment of unresectable or metastatic HCC in China. In the sensitivity analysis, most of the ICERs fell into the southeast quadrant and above the three times GDP per capita WTP threshold in China, which suggested a relatively low probability of being cost effective and confirmed the robust results of the PSA. However, replacement of the branded price of sorafenib in the model led to a positive result.

The Chinese National Medical Products Administration (NMPA) approved donafenib as an innovative first-class drug for the first-line treatment of unresectable or metastatic HCC patients who had not received systemic treatment as of June 2021. The approval of donafenib was based on a large RCT (ZGDH3) conducted across 37 centers in China, and its efficacy and safety results were similar to the actual situation of patients with advanced HCC in China (5). Although similar PFS outcomes between donafenib and sorafenib were observed in ZGDH3 and via extrapolation, a significantly longer OS duration led to more QALYs gained with donafenib.

When conducting a cost-effectiveness analysis of anticancer drugs, due to limited follow-up results from RCTs, long-term extrapolation is needed to simulate the development of disease (25–27). However, different fitting distributions of the survival data might impact the results, and extrapolation will increase the uncertainty of long-term modeling. Therefore, a scenario analysis using only original survival data from the ZGDH3 study without extrapolation was performed to verify the uncertainty in this study. A higher ICER was generated in the scenario analysis, which was in line with the base-case results not being cost effective.

To increase access to high-value, high-price drugs and reduce the economic burden of patients, price negotiation has been required since 2017 by the National Healthcare Security Administration (NHSA) when the pharmaceutical manufacturer of a new drug applies for coverage on the National Reimbursement Drug List (NRDL) in China (28). Donafenib will join the 2021 NRDL negotiation. For branded sorafenib, an agreement with NHSA was reached in 2017 for price reduction, and the agreement was renewed in 2019. With the introduction of generic drugs for sorafenib, two such domestic generic drugs won the bidding with very low prices (approximately one-third of branded price) via Fourth Volume-based Procurement in 2021. The government promises that they will provide these two domestic generic drugs to 60% of the national market of public hospitals within the next year. Therefore, the mean price of VBP was set in the base-case analysis, and the branded price was explored in the scenario analysis. Donafenib was not cost effective when compared to the low price of generic sorafenib but had a positive result when compared to the branded price of sorafenib.

This study also has some limitations. First, due to the lack of domestic utility for patients with advanced HCC in China, this study used utility data from lenvatinib vs. sorafenib in the first-line treatment of patients with advanced HCC in the NICE trial (TA551) (11), which is the most suitable data at present. Considering that the utility of different states has a great impact on the ICER, it is necessary to update the current model results when local utility data become available in the future. Second, since the ZGDH3 study did not specify the treatment therapies administered to patients after disease progression, this study assumed that patients in the donafenib and sorafenib groups would receive the same subsequent treatment after progression occurred. The subsequent treatment cost was therefore adopted from a previous cost-effectiveness analysis for Chinese patients with advanced HCC in 2020 (17). Third, the survival data were derived and reconstructed from RCTs, which may not reflect real-world clinical practice in China. Forth, without the access of original individual patient data of the clinical trial, subgroup analysis to demonstrate heterogeneity of patients' characteristics cannot be performed.

## Conclusion

Donafenib provides a new option for advanced HCC patients; however, the results of this economic evaluation suggest that compared with sorafenib, it unlikely to be cost effective for the first-line treatment of unresectable or metastatic HCC in China at the current price. A reduction in price or comparison with branded sorafenib will increase the probability of it being cost effective.

## Data Availability Statement

The datasets presented in this study can be found in online repositories. The names of the repository/repositories and accession number(s) can be found in the article/Supplementary Material.

## Author Contributions

TZ and RM developed the model to conduct the cost-effectiveness analysis. RM and YC reconstructed the individual patient data and identified the cost and utility parameters. RM, HH, and YQ checked the data and wrote the manuscript. TZ revised the manuscript. All authors reviewed and approved the final version.

## Conflict of Interest

The authors declare that the research was conducted in the absence of any commercial or financial relationships that could be construed as a potential conflict of interest.

## Publisher's Note

All claims expressed in this article are solely those of the authors and do not necessarily represent those of their affiliated organizations, or those of the publisher, the editors and the reviewers. Any product that may be evaluated in this article, or claim that may be made by its manufacturer, is not guaranteed or endorsed by the publisher.
