# Supplementary material for: The Cost Effectiveness of Donafenib Compared With Sorafenib for the First-Line Treatment of Unresectable or Metastatic Hepatocellular Carcinoma in China
Source: Front Public Health. 2022 Mar 31;10:794131. doi: 10.3389/fpubh.2022.794131 (PMC9008355; doi:10.3389/fpubh.2022.794131)
Supplement: Supplementary file 1 [file Data_Sheet_1.pdf]

Supplementary Materials

The Cost Effectiveness of Donafenib Compared with Sorafenib for the First-line Treatment of Unresectable or Metastatic Hepatocellular Carcinoma in China

Supplementary Table S1. The AIC and BIC of alternative distributions of KM curves

	**KM curve**	**Exponential**	**Gamma**	**Gompertz**	**Weibull**	**Loglogistic**	**Lognormal**
AIC	DONOS	1941.88	1927.31	1943.88	1934.36	1904.85	**1895.89**
	DONPFS	1365.01	1342.73	1361.84	1358.90	1273.06	**1266.95**
	SOROS	2051.55	2025.32	2050.67	2033.48	2010.20	**2006.98**
	SORPFS	1478.43	1450.28	1471.61	1471.59	**1351.03**	1356.90
BIC	DONOS	1945.68	1934.89	1951.46	1941.95	1912.44	**1903.48**
	DONPFS	1368.80	1350.32	1369.43	1366.48	1280.65	**1274.54**
	SOROS	2055.35	2032.92	2058.27	2041.08	2017.81	**2014.58**
	SORPFS	1482.23	1457.88	1479.22	1479.20	**1358.63**	1364.51

AIC Akaike information criterion, *BIC* Bayesian information criterion, *KM* Kaplan-Meier, *DON*, donafenib, *SOR* sorafenib, *OS* overall survival, *PFS* progression-free survival.

Supplementary Table S2. The survival function formula and parameter value of optimal distribution of KM curve.

KM curve	optimal distribution	Survival function formula	value of the parameter	
			μ	δ
DONOS	Lognormal		2.53124	1.0034388
SOROS	Lognormal	S(t)=1-φ[(log(t)-μ)/σ]	2.376404	0.9443653
DONPFS	Lognormal		1.336398	0.8439057
			λ	γ
SORPFS	Log-logistic	S(t)=1/[1+(λt)^(1/γ)]	3.2420	2.2093

KM Kaplan-Meier, *DON* donafenib, *SOR* sorafenib, *OS* overall survival, *PFS* progression-free survival.

Supplementary Table S3. Median OS and PFS of reconstructed KM curves.

KM curve	median value of reconstructed KM curve	median value of KM curve in trial
DONOS	12.2 m	12.1 m
SOROS	10.2 m	10.3 m
DONPFS	3.6 m	3.7 m
SORPFS	3.58 m	3.6 m

KM Kaplan-Meier, *DON* donafenib, *SOR* sorafenib, *OS* overall survival, *PFS* progression-free survival, *m* month.
